# Shifts in the gut microbiota of sea urchin *Diadema antillarum* associated with the 2022 disease outbreak

**DOI:** 10.3389/fmicb.2024.1409729

**Published:** 2024-07-29

**Authors:** Juliana M. Ruiz-Barrionuevo, Elif Kardas, Ruber Rodríguez-Barreras, Marcos A. Quiñones-Otero, Claudia P. Ruiz-Diaz, Carlos Toledo-Hernández, Filipa Godoy-Vitorino

**Affiliations:** ^1^Instituto de Ecología Regional (IER), Universidad Nacional de Tucumán (UNT)-Consejo Nacional de Investigaciones Científicas y Técnicas (CONICET), Tucumán, Argentina; ^2^Facultad de Ciencias Naturales e Instituto Miguel Lillo, Universidad Nacional de Tucumán (UNT), Tucumán, Argentina; ^3^Department of Microbiology, University of Puerto Rico, School of Medicine, San Juan, PR, United States; ^4^Department of Biology, University of Puerto Rico, Rio Piedras campus, San Juan, PR, United States; ^5^Department of Biology, University of Puerto Rico, Mayagüez campus, Mayagüez, PR, United States; ^6^Department of Biology, University of Puerto Rico at Bayamón, Bayamón, PR, United States; ^7^Planning Department, University of Puerto Rico, Río Piedras Campus, San Juan, PR, United States; ^8^Sociedad Ambiente Marino, San Juan, PR, United States

**Keywords:** *Diadema antillarum*, sea urchin, gut microbiota, outbreak, 16S rRNA, Puerto Rico

## Abstract

**Introduction:**

In recent decades, Caribbean coral reefs have lost many vital marine species due to diseases. The well-documented mass mortality event of the long-spined black sea urchin *Diadema antillarum* in the early 1980s stands out among these collapses. This die-off killed over 90% of *D. antillarum* changing the reefscape from coral to algal-dominated. Nearly 40 years later, *D. antillarum* populations have yet to recover. In early 2022, a new mortality event of *D. antillarum* was reported along the Caribbean, including Puerto Rico.

**Methods:**

This study identifies the gut microbiota changes associated with the *D. antillarum* during this mortality event. It contrasts them with the bacterial composition of gut samples from healthy individuals collected in 2019 by using 16S rRNA sequencing analyses.

**Results:**

Notably, the die-off group’s core microbiome resembled bacteria commonly found in the human skin and gut, suggesting potential anthropogenic contamination and wastewater pollution as contributing factors to the 2022 dysbiosis. The animals collected in 2022, especially those with signs of disease, lacked keystone taxa normally found in *Diadema* including *Photobacterium* and *Propionigenium*.

**Discussion:**

The association between human microbes and disease stages in the long-spined urchin *D. antillarum*, especially in relation to anthropogenic contamination, highlights a complex interplay between environmental stressors and marine health. While these microbes might not be the direct cause of death in this species of sea urchins, their presence and proliferation can indicate underlying issues, such as immune depletion due to pollution, habitat destruction, or climate change, that ultimately compromise the health of these marine organisms.

## 1 Introduction

Marine ecosystems, once considered vast and resilient, now face the escalating challenge of pandemic diseases that disrupt their delicate balance (Bidegain et al., 2016). Reports of large-scale episodic events resulting in mass mortalities among marine organisms have risen since the latter part of the previous century (Hayes et al., 2001; Bidegain et al., 2016). Pandemic diseases in marine ecosystems stem from a complex interplay of environmental stressors, human activities, and climate change (Abbass et al., 2022; King et al., 2023). Thus, it is important to understand the underlying causes and mechanisms behind these disease outbreaks to effectively predict and manage them, just as we do for land-based systems. Over the years, marine diseases, including animals from aquaculture, have long been associated with higher water temperature, eutrophication, injuries, and infections (Brink et al., 2019; Chi et al., 2022).

Disease outbreaks with a profound impact on marine communities have been associated with anthropogenic stressors such as climate change (Burge et al., 2014; Priya et al., 2023). In the last 50 years, there have been notable decreases in echinoids, specifically linked to illnesses (Feehan and Scheibling, 2014; Roth et al., 2024). One remarkable example was the extensively documented mass mortality event affecting the long-spined black sea urchin *Diadema antillarum* (Philippi, 1845) in the early 1980s (Lessios, 2016). This event had far-reaching consequences on reef communities in the Caribbean, leading to significant shifts in the structure and functioning of coral reef ecosystems in the region (Carpenter, 1990; Manuel et al., 2021). The mass mortality event of *D. antillarum* triggered a surge of interest in studying urchins, leading to valuable discoveries in the field of their ecology, taxonomy, reproduction, and molecular analysis (Williams et al., 2013). This newfound enthusiasm has also contributed to groundbreaking studies that use powerful sequencing techniques to revolutionize our understanding of the prokaryotes associated with echinoderms (Loudon et al., 2023; Marangon et al., 2023; Rodríguez-Barreras et al., 2023a). For instance, a recent study performed the first-ever characterization of the microbiota linked with four Caribbean sea urchin species (Rodríguez-Barreras et al., 2021). The findings revealed a distinct partitioning of microbiota based on the specific feeding niches of each sea urchin species, with sea urchins from the reef environments showing a prevalence of sulfate-reducing bacteria, whereas those inhabiting seagrass habitats exhibited a dominance of Planctomycetes and Cyanobacteria.

In 2022, a new, scuticociliate-associated mortality event ravaged *D. antillarum* populations across several Caribbean islands (Hylkema et al., 2023). Initial reports surfaced in the U.S. Virgin Islands, but similar mortalities soon spread to other islands (Hylkema et al., 2022; Rodríguez-Barreras et al., 2023b). Afflicted *D. antillarum* exhibited distinctive disease signs, including sea urchins venturing beyond their usual shelters during mid-day, inability to securely attach to the substrate, sluggish spine movement, and noticeable spine loss (Hylkema et al., 2023). A recent article evidenced that dysbiosis could be associated with pathogenesis, a direct consequence of the occurrence of the scuticociliate species similar to *Philaster apodigitiformis* (Hewson et al., 2023). These parasitic (ciliate) diseases affecting these sea urchins can have significant ecological impacts and include changes in the physiological balance of the animal’s microbiota. This study leverages samples collected before (i.e., 2019) and during the initial stage of the 2022 die-off emergency to investigate its impact on the gut prokaryotic communities of *D. antillarum*. We hypothesize that there would be changes in the microbiota among the animals collected previous to and during the 2022 die-off event. Understanding the relationship between microbial communities and sea urchin health is crucial for conservation efforts.

## 2 Materials and methods

### 2.1 Sea urchin collection and dissection

Two sets of *D. antillarum* individuals (*n* = 23) were collected in their reef habitats in 2019 and early 2022.

**Figure 1:**
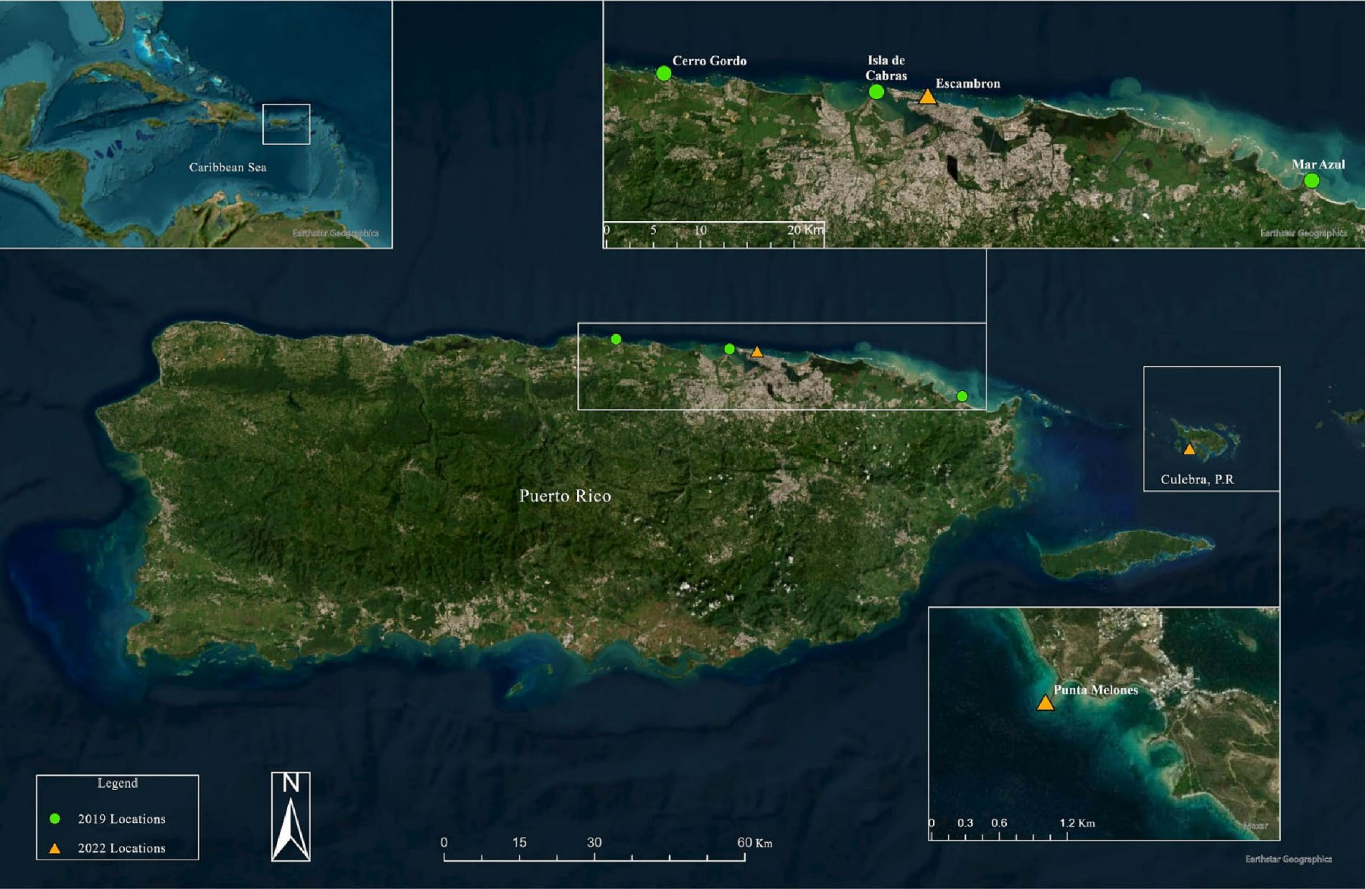
Surveyed sites in 2019 and 2022 for the analysis along the northern coasts of Puerto Rico and Culebra Islands. The 2019 locations represented in green color circles are Cerro Gordo (CGD), Mar Azul in Luquillo (LUQ), and Isla de Cabras en Cataño (CAT). The 2022 locations represented in orange triangles are Escambron (ESC), Punta Melones in Culebra (CUL). Produced by Marcos A. Quinones-Otero. Image credit: Esri, Maxar, Earthstar Geographics, and the GIS User Community.

The first dataset corresponds to the 2019 sample collection derived from Rodríguez-Barreras et al. (2021) and included 15 sea urchins (hereafter referred to as “2019_healthy group”) collected at three shallow-water sites (1–2 m water depth) from the Northeastern coast of Puerto Rico: 4 sea urchins in Cerro Gordo located in Vega Baja (CGD, 18°29′05.81″N, −66°20′20.23″W), 6 in Isla de Cabra in Cataño (CAT, 18°28′26.32″N, −66°08′18.82″W), and 5 in Luquillo (LUQ; 18°23′15.01″N, −65°43′11.26″W) (Table 1). During the 2022 collection, 8 long-spined black sea urchins were collected at two sites, Playa El Escambrón in San Juan (ESC; 18°27′57.9″N, −66°05′09.4″W) and in Punta Melones in Culebra (CUL; 18°18′14.2″N, −65°18′39.9″W; Figure 1 and Table 1). At ESC, three individuals exhibited signs of disease (hereafter referred to as “2022_diseased group”), including being outside their shelters during mid-day, difficulty attaching to the substrate, sluggish spine movement when touched, and autotomy (spine loss) (Rodríguez-Barreras et al., 2023a,b). Three other individuals showing no diseased signs were also collected at ESC (hereafter named the “2022_healthy group”). In CUL, the two collected individuals were diseased. All specimens (considered diseased or visually healthy) were placed in separate plastic bags filled with seawater, temporarily put in a foam cooler, and transported to the laboratory. We recognize the low sample size. The number of samples collected during the 2022 outbreak emergency was limited by two factors: permit restrictions from the Puerto Rico Department of Environmental and Natural Resources, and the availability of samples at the time of collection. This study received approval from the Department of Natural and Environmental Resources of Puerto Rico (Permit #: DRNA-2022-IC-026, O-*VS*-PVSlS-SJ-01291-06052022). The local permit at the collection emergency time indicated the collection of only six individuals at two healthy sites and a maximum of nine individuals in sites with a disease report. The selection of sites in 2022 was based on (1) the site similarities regarding the structural complexity of their habitats, (2) the presence of *D. antillarum* populations before the 2022 pandemic, and (3) reports on social media pointed to outbreaks in the selected sites, that is, Culebra and Escambron. Other sites surveyed by Rodríguez-Barreras et al. (2021) were visited in 2022; however, no *D. antillarum* individuals were sighted at the time of the surveys.

**Table 1:** Description of the collected samples of the sea urchin *Diadema antillarum* used in this study per site, pre-die-off (2019), and die-off (2022).

Site	Year	Health status
Cerro Gordo (CGD)	2019	Healthy
Luquillo (LUQ)	2019	Healthy
Cataño (CAT)	2019	Healthy
Culebra (CUL)	2022	Diseased
Escambron (ESC)	2022	Diseased
Escambron (ESC)	2022	Healthy

Sea urchin collection sites and their corresponding abbreviation (Cerro Gordo (CGD), Luquillo (LUQ), Cataño (CAT), Culebra (CUL), and Escambron (ESC)), year of collection (2019, 2022) and health status (healthy, diseased).

The animal euthanization and dissection protocol was approved by the University of Puerto Rico Medical Sciences Campus IACUC protocol (IACUC #: A5301118). Euthanization consisted of placing specimens in 100 mL glass beakers with 25 mL of transporting seawater allowing them to acclimate for a minimum of 10 min, or until they adhered to the beaker walls. For sedation, a 25 mL sterile solution of 20 mM of MgCl_2_ was used, a common procedure used in aquaculture research (Arafa et al., 2007). We monitored the experimentally induced sedation until all specimens detached from the beaker walls. Individuals were carefully transferred to a metal tray and exposed to an ultra-low temperature of −80°C for 10 min (thermal shock) before dissection. Fecal pellets were carefully collected from the animal’s guts.

### 2.2 DNA extraction

DNA from fecal pellet samples was extracted using the PowerSoil Pro Kit (QIAGEN LLC, Germantown Road, Maryland, United States) following the manufacturer’s instructions. A Qubit® dsDNA HS assay kit (High Sensitivity) (Waltham, Massachusetts, United States) was used to assess DNA concentrations of purified extracts. Sequencing was outsourced at Argonne National Laboratory (Illinois, United States) using Illumina MiSeq with the 2 × 250 bp paired-end sequencing kit. The DNA was normalized to 4 nM during 16S rRNA gene library preparation. The facility followed the Earth Microbiome Project standard protocols^1^ using universal bacterial primers: 515F (5′GTGCCAGCMGCCGCGGTAA3′) and 806R (5′GGACTACHVGGGTWTCTAAT3′) to amplify the hypervariable region V4 of the 16S ribosomal RNA gene (~291 bp) (Caporaso et al., 2012). Amplicons were quantified using PicoGreen (Invitrogen) and a plate reader (Infinite^®^ 200 PRO, Tecan). Once quantified, the volumes of each of the products were pooled into a single tube so that each amplicon was represented in equimolar amounts. This pool was then cleaned up using AMPure XP Beads (Beckman Coulter) and then quantified using a fluorometer (Qubit, Invitrogen). The obtained demultiplexed reads and the corresponding metadata were uploaded to QIITA Study ID 14784. The raw data are available at the European Nucleotide Archive Project (ENA) under the access numbers EBI: ERP123720 https://www.ebi.ac.uk/ena/browser/view/PRJEB40117 (2019 animals) and ERP155238 https://www.ebi.ac.uk/ena/browser/view/PRJEB70304 (2022 animals).

### 2.3 Sequence processing and statistical analysis

#### 2.3.1 Quality control of the reads and standard microbiota analyses

Sample read pre-processing was done using QIITA (version 2022.09). The 16S rRNA genes were trimmed to 250 bp and classified using the SILVA database’s closed reference approach (Quast et al., 2013). Sequences corresponding to chloroplasts, mitochondria, and singletons (*n* < 5) were removed from downstream analyses.

Rarefaction was done at 923 reads when comparing all animal samples collected in 2019 and 2022, ending in the removal of two diseased samples at ESC. To test for differences at other rarefaction levels and include most samples, we also performed rarefaction considering 539 reads and analyzed the dataset without rarefaction (Supplementary Figures S1, S2). These different analyses found that despite rarefaction, bacterial composition remained the same. Rarefaction is a standardized analysis that uses the same number of sequencing reads across all samples, reducing it to a common threshold, which allows for fair comparisons despite variations in sequencing depth or effort. We acknowledge that this is the subject of considerable debate in the field which is beyond the scope of this article (McMurdie and Holmes, 2014), although other authors show that rarefaction guarantees that subsequent permutation tests properly control the Type I error (Hong et al., 2022) and are still widely used in the scientific community.

A meta-analysis was performed in QIITA (version 2022.09) using two datasets: (1) gut microbiota of *D. antillarum* samples collected at CGD, CAT, and LUQ in 2019 (QIITA Project ID: 12668) and (2) *D. antillarum* gut microbiota and coelomic samples collected in ESC and CUL in the die-off event in 2022 (QIITA Project ID: 14784). For both datasets (using only gut microbiota samples), we used the same sequencing facility, the same parameters for quality control, and a closed reference OTU method using the SILVA database (Quast et al., 2013). The *biom* file of the joint meta-analyses was downloaded from QIITA for downstream analyses using QIIME2 (Caporaso et al., 2010).

The downstream processes of alpha- and beta-diversity estimates using the resulting *species* table (.*biom* format) were done as previously reported (Rodríguez-Barreras et al., 2021; Ortiz et al., 2022; Ruiz Barrionuevo et al., 2022). We used the indices of Chao1 (richness; Chao and Chiu, 2016) and Shannon (diversity index; Shannon, 1948) to assess alpha-diversity comparisons between years (2019 vs. 2022) and collection sites. To evaluate beta-diversity according to year, collection sites, and health status, we plotted the Bray–Curtis dissimilarity index using non-metric multidimensional scaling (NMDS) with samples colored according to year and diseased state and shaped according to the collection site. Beta-diversity statistical tests including PERMANOVA (Anderson, 2001), PERMDISP (McArdle and Anderson, 2001), and ANOSIM (Clarke, 1993) were applied to quantify dissimilarity between all comparison groups (health status per collection year, and collection site). PERMANOVA and ANOSIM analyses were both applied to compare the dispersion of the Bray–Curtis dissimilarity index in the NMDS. A PERMDISP analysis was used to test for the PERMANOVA assumption of homogeneity of multivariate variances. Beta-diversity plots were built using non-metric multidimensional scaling (NMDS) (Clarke, 1993). The alpha- and beta-diversity plots and the compositional taxa barplots were built in *R* with *ggplot2* (Wickham, 2009) using a colorblind-friendly palette (Sweet, 2020).

Finally, the compositional heatmap (considering the site of collection) and heat tree compare overall taxa significantly changing in pre-die-off and die-off groups. This analysis was done with the MicrobiomeAnalyst online tool (Dhariwal et al., 2017) using standard parameters, including a low-read filter of 4, considering only reads present in at least 20% of the samples (20% core taxa), and total sum scaling without transformation.

### 2.4 Microbiome multivariable association with linear models and putative biomarker taxa identification

Bacterial taxa associated with the health status per year, per collection site, were identified using Multivariate Analysis by Linear Models (MaAsLin2) (Mallick et al., 2021). MaAsLin is a comprehensive R package for determining the multivariable association between metadata and microbial features. After pattern matching to allow flexible capitalization of the correction argument, “fdr” resolves to “BH” in p.adjust and gives a list of *p*-values and adjusted *q*-values which are plotted for <0.05 in a heatmap. The MaAsLin2 model included the health status (healthy vs. diseased) correcting for the collection years (2019 vs. 2022) and sites (ESC, CAT, CGD, LUQ, and CUL). In addition, we compared the diseased animals (collected in 2022) with those that resembled healthy (collected in 2019), correcting for sites.

### 2.5 Inference of bacterial metabolic functions

Metabolic inferences of functions, including Sulfate Respiration, Respiration of Sulfur Compounds, and human-associated taxa, were assigned to the bacterial taxa (genera) using FAPROTAX 1.2.6 (Louca et al., 2016). The FAPROTAX database was used to assign the functional activity of bacteria associated with sea urchins’ gut pellets, rather than other functional prediction databases, such as PICRUST2 or TaxForFun, as the former has been developed specifically through actual functional activity from global ocean bacteria (Louca et al., 2016). We selected metabolic functions with only positive values and those with a standard deviation higher than 0.01 to compare (1) the health status considering the year (2019_healthy, 2022_healthy, and 2022_diseased) and (2) the different collection sites per year (“2019_CAT,” “2019_CGD,” “2019_LUQ,” “2022_CUL,” and “2022_ESC”).

## 3 Results

### 3.1 *Diadema antillarum* samples

A total of 572,863 reads were included in the main analyses, and rarefaction was done at 923 reads (Supplementary Table S1). After rarefaction, and having removed two samples from ESC due to low-read depth, we analyzed 21 samples from 15 animals collected in 2019, pre-die-off, and 6 animals collected in 2022 during the die-off event. Additional supplementary analysis was done both with a rarefaction level of 539 reads as well as without rarefaction to include all samples. The richness plateau was similar between the different rarefaction values, that is, rarefaction at 923 and 539 reads (Supplementary Figures S1B,C, respectively). Despite rarefaction levels and even when we did not rarefy, we found similar patterns in bacterial composition and diversity among samples (Supplementary Figures S1, S2).

### 3.2 Bacterial diversity and composition: pre-die-off vs. die-off

**Figure 2:**
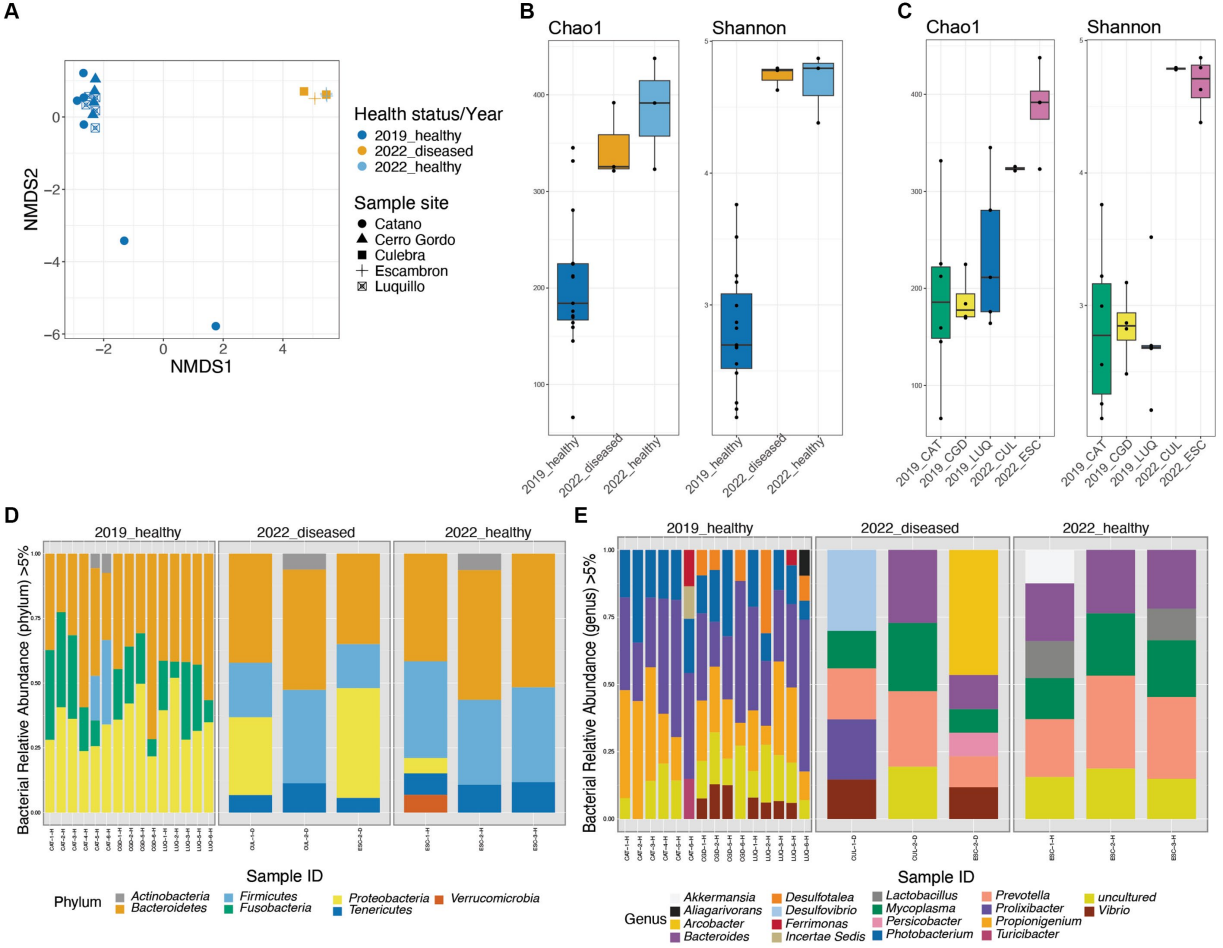
Diversity plots and taxonomic composition of *Diadema antillarum* gut bacterial communities in 2019 (healthy and pre-die-off) and 2022 (healthy and diseased during the die-off). Beta-diversity Non-metric multidimensional scaling (NMDS) derived from Bray-Curtis distances **(A)**. Alpha-diversity box-plots from Chao1 and Shannon’s indices comparing the three groups of healthy and diseased animals collected in 2019 and 2022 revealed significant differences between the 2019_healthy group and the 2022 group (Chao 1 *p*-value = 0.0021, Shannon *p*-value = 0.0061, respectively). **(B)**. Alpha-diversity boxplots from Chao1 and Shannon’s indices comparing the different sites of collection showed no significant differences (cf. Supplementary Table S3) **(C)**. Taxonomic barplots at the phylum **(D)** and genus levels **(E)**, panels are dividing the three groups of healthy and diseased animals collected in 2019 and 2022. The reads were rarefied at 923 for this analysis.

The main variables of our study were the period of collection pre-die-off vs. die-off, animal status (healthy or diseased animals), and the collection sites. The beta-diversity NMDS derived from the Bray–Curtis index showed a clear separation between the fecal bacterial communities of the 2019 and 2022 groups (despite collection sites and disease status) (PERMANOVA *p*-value < 0.01 and ANOSIM *p*-value < 0.01). However, the spread between samples in both years was not significantly different (PERMDISP *p*-value > 0.05) (Figure 2A). Shannon and Chao alpha-diversity indices revealed a significantly higher microbial diversity in the fecal pellets from the 2022 group (regardless of the healthy status and the site of collection) than the 2019_healthy group (*p*-value = 0.0021, *p*-value = 0.0061, respectively; Figure 2B and Supplementary Table S2). The alpha-diversity comparison according to collection sites showed the same pattern of higher diversity in 2022 samples (*p*-value = 0.0128 for Shannon, *p*-value = 0.0355 for Chao; Figure 2C and Supplementary Table S2). However, both alpha-diversity indices revealed no significant differences between the 2022_healthy and 2022_diseased groups, regardless of the collection site (*p*-value > 0.05; Figure 2B and Supplementary Table S3). Due to the emergency situation, and therefore, the lack of the rigorous planned nature of the study, environmental parameters were not measured. However, the similarity among animal microbiota across sites, with only ESC being in a highly populated area among the 2022 samples, suggests minimal environmental variation between sites. This leads us to believe that any environmental differences that may have existed had a negligible impact on the animal’s microbial communities.

The taxonomic composition also differed between the 2019_healthy group and the 2022 groups (whether visually healthy or diseased and independently of the site of collection) (Figures 2D,E).

At the phylum level, we found a high abundance of Bacteroidetes across all samples. Firmicutes were the second most abundant phylum in both groups of 2022 (whether visually healthy or diseased and sites), and Proteobacteria was more dominant in the diseased animals (Figure 2D; Supplementary Figure S2). In contrast, the 2019_healthy group displayed a higher abundance of Proteobacteria and Fusobacteria, with Fusobacteria being absent in both groups of 2022 (whether visually healthy or diseased) (Figure 2D). The bacterial composition whether at the phylum (Figure 2D; Supplementary Figures S2, S3 or genus level Figure 2E; Supplementary Figures S2–S5) of sea urchins collected in 2022 did not differ between Culebra (CUL) and Escambron (ESC), whether they were visually healthy or diseased.

At the genus level, both groups of 2022 (whether visually healthy or diseased) were characterized by a dominance of *Prevotella*, *Mycoplasma*, *Bacteroides*, and the presence of *Lactobacillus*, *Vibrio*, *Akkermansia*, and *Desulfovibrio*. In contrast, the 2019_healthy group harbored a higher abundance of *Prolixibacter*, *Propionigenium*, and *Photobacterium* (Figure 2E). To note, within individuals from both groups of 2022 (whether visually healthy or diseased), there were some differences in taxonomic composition between sites, yet with few changes in the relative abundance of human-associated bacteria that included *Gardnerella*, *Akkermansia*, and *Bacteroides* and were clustered in the PCoA plot (Supplementary Figure S5A). However, two diseased individuals, from ESC and CUL, showed a distinctive abundance of *Prolixibacter*, *Desulfovibrio*, and *Photobacterium*, which are bacteria commonly found in the healthier group. These individuals clustered in a different group than the above (Supplementary Figures S5A,B). This contrast in bacterial dominance may suggest that the diseased individuals were at different stages of the disease.

### 3.3 Bacterial composition changes according to multivariate associations with linear models

**Figure 3:**
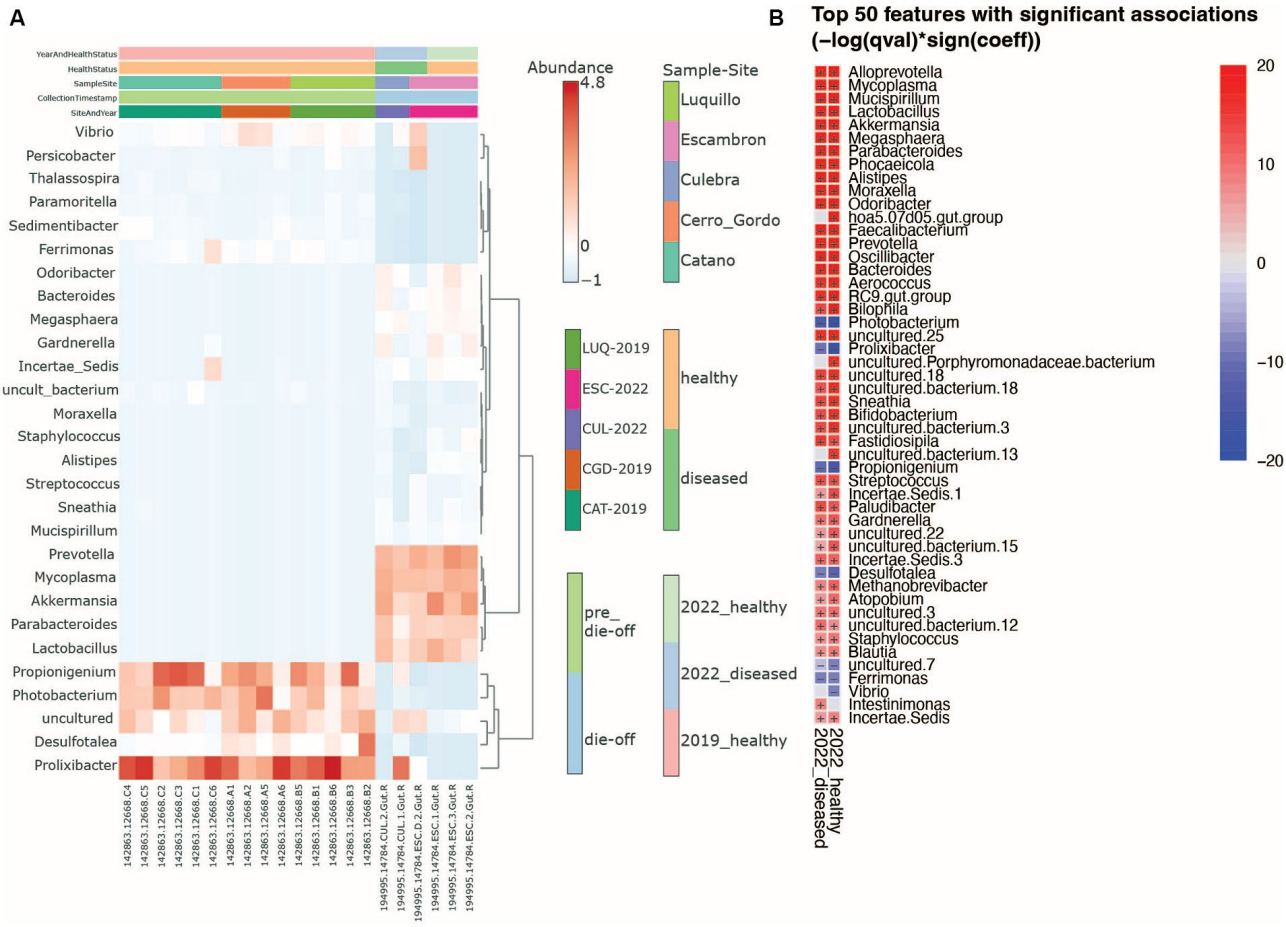
Heatmaps show genus-level changes in the microbiota. **(A)** Overall differences between sea urchin’s health year and collection sites. **(B)** Heatmap resulting from the bacterial Multivariate Analysis by Linear Models (MaAsLin) comparing healthy and diseased animals in 2022 using as reference the healthy animal microbiota from 2019 samples. Red (+) indicates a positive association while blue (−) a negative one. The *p*-value threshold is set at 0.05.

As we mentioned earlier, there were differences in bacterial composition mainly between the 2019_healthy group and 2022 groups (whether visually healthy or diseased): *Prolixibacter*, *Propionigenium,* and *Photobacterium* were among the most prevalent genera in sea urchin individuals from the 2019_healthy group, whereas *Prevotella*, *Lactobacillus*, *Akkermansia*, *Parabacteroides*, and *Mycoplasma* were more prevalent in the microbiota of the 2022 groups (Figure 3A). We used MaAsLin (Microbiome Multivariate Association with Linear Models) to identify the top 50 significantly different genera between the 2019_healthy group and the 2022 groups (whether visually healthy or diseased). We found that animals collected in 2022, whether visually healthy or diseased, had more human-associated taxa compared to the 2019_healthy group, for example, *Prevotella*, *Akkermansia*, or *Mycoplasma* with a loss in *Propionigenium*, *Ferrimonas*, *Vibrio*, and *Photobacterium* as in 2019 (Figure 3B). Finally, the heat tree (Supplementary Figure S4) displayed the changes in composition between pre-die-off and die-off groups taking into account both taxonomic hierarchy and differential abundance, finding the same human-derived bacteria augmented in the die-off period (shown in blue as reduced in pre-die-off samples).

### 3.4 Microbial functional assemblages predicted by FAPROTAX

**Figure 4:**
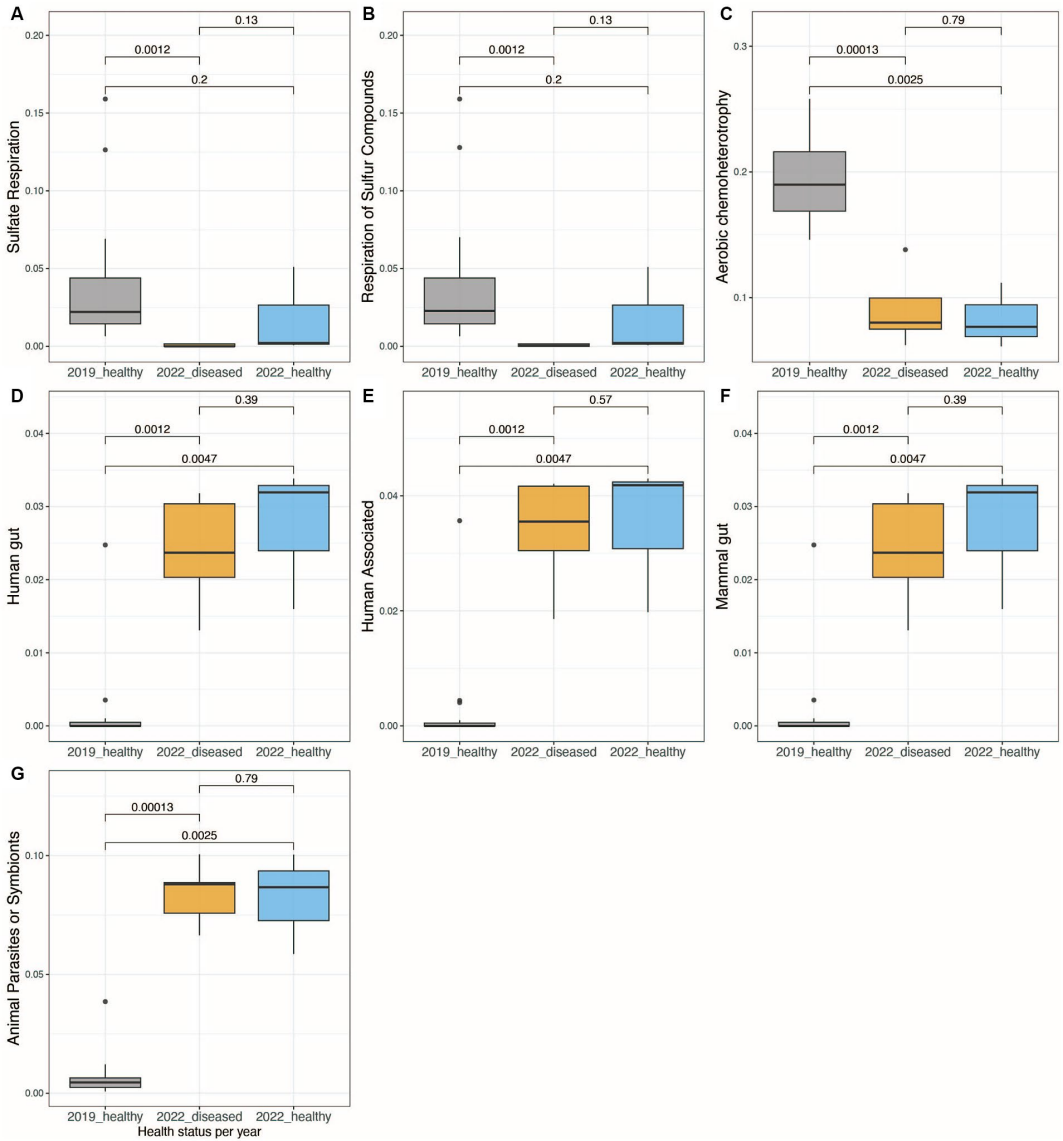
Boxplots showing the significant differences in bacterial functional activity between the three groups of healthy and diseased animals collected in 2019 and 2022, using Functional Activity of Prokaryote taxa (FAPROTAX) database **(A–G)**. The functions shown are those that showed a standard deviation higher than 0.01 when comparing the relative abundance of functional, active taxa between samples..

Using the FAPROTAX database, bacterial communities from the long-spined black sea urchin *D. antillarum* fecal pellets were characterized for their inferred metabolic and ecological functions. When comparing the 2019-healthy group sea urchins with the 2022 groups (whether visually healthy or diseased), there was a significant shift in their metabolic and ecological functions (Kruskal-Wallis test, *p*-value < 0.05, Figures 4A–G, Supplementary Figures S6A–H). When comparing the two groups of 2022, i.e., visually healthy versus diseased, no significant differences in metabolic and ecological functions were found (Kruskal-Wallis test, *p*-value > 0.05 for all functions, Figures 4A–G, Supplementary Figures S6A–H). While the 2022-few bacterial communities doing sulfate and sulfur compounds respiration and aerobic chemoheterotrophy (Figures 4A–C, Supplementary Figures S6A–H), they exhibited bacterial communities characteristic of human, human-gut, mammal guts, and animal parasites or symbionts (Figures 4A–C, Supplementary Figures S6A–H). The 2022-healthy group did not appear to completely recover from this bacterial metabolic and functional shift. Indeed, this group displayed none to few sulfate and sulfur compounds respiration and aerobic chemoheterotrophy activities but was still characterized by bacterial communities from human, human gut, mammal guts, and animal parasites or symbionts (Kruskal–Wallis test, *p*-value < 0.05, Figures 4D–G). When comparing the sea urchin pellets from the different collection sites per year (“2019_CAT,” “2019_CGD,” “2019_LUQ,” “2022_CUL,” and “2022_ESC”), there was some significant shift in the metabolic and ecological functions between sites, but the most significant shift of metabolic and ecological functions concerned human gut, human-associated, mammal-associated, and parasites and symbionts taxa, which were higher in 2022 than 2019 (Kruskal–Wallis test, *p*-value < 0.05, Supplementary Figures S6D–G) but not between sites from each year (Kruskal–Wallis test, *p*-value > 0.05, Supplementary Figures S6D–G).

## 4 Discussion

Diseases impact every aspect of life, including cells, tissues, individuals, populations, communities, and even ecosystems, and there is no exception for marine species (Sweet, 2020). Disease prevalence in marine organisms is said to be rising globally (Tracy et al., 2019). For many species, large-scale disease epidemics have been linked to anthropogenic stressors such as climate change, representing a serious concern for marine organisms (Sweet et al., 2016). Studies have recognized the gut microbiome as an important driver of various metabolic processes in the host. However, recent publications have targeted mostly human diseases and farming species (Milan et al., 2019; Holt et al., 2021). In addition, when the focus is on dysbiosis, most studies emphasize the consequences of microbiome dysbiosis in the immune system of the host and not how pathogens can cause changes in the host microbiome (Toledo-Hernández and Ruiz-Diaz, 2014; Egan and Gardiner, 2016; Infante-Villamil et al., 2021). Significant changes in the host microbiota have also been reported in other studies. For example, a recent study conducted on the sea urchins *Holopneustes purpurascens* and *Heliocidaris erythrogramma* found significant differences between the bacterial diversity of healthy and diseased tissue samples (Sweet et al., 2016).

As already discussed by other colleagues, disease signs observed in *D. antillarum* during the 2022 outbreak event mirrored those of the past die-off including delayed spine movement, spine loss, and necrosis (Hylkema et al., 2023). The extent of the die-off remains undetermined although an important study has suggested it could be due to an invasion by *Philaster apodigitiformis* (Hewson et al., 2023). This microbiota study reveals a significant shift in the gut bacterial communities of *D. antillarum* during the die-off event, supported by high bacterial diversity and different composition analyses as compared to the microbiota of the 2019 *Diadema* individuals. This microbiota dysbiosis could be associated with pathogenesis and directly consequenced by *Philaster apodigitiformis* (Hewson et al., 2023) as animals collected in 2022 had signs compatible with scuticociliatosis. Despite the fact that *P. apodigitiformis* can cause disease, not all ciliates cause dysbiosis in *D. antillarum*. In fact, other ciliates of the genus *Metopus*, *Anophrys*, *Cryptochilidium*, *Cohnilembus,* and *Cyclidium* have been found in gut samples of *D. antillarum* with no evidence of pathological conditions (Randall et al., 1964). Moreover, we hypothesize that the differences found within die-off samples collected in 2022 in Puerto Rico could be explained by two major factors: the potential differences of the anthropogenic impacts between surveyed sites, and the stage of disease progression. Although formal environmental assessments were not conducted at the sampling sites, visual observations revealed varying levels of human influence, with ESC exhibiting higher levels of anthropogenic impact among all surveyed locations. Moreover, the core microbiome from the 2022 healthy and diseased groups was dominated by bacteria commonly found in the human gut (Thursby and Juge, 2017), suggesting sewage pollution as a possible driver of the dysbiosis observed in these groups. The genus-level taxonomic changes indicate that ESC samples evidence a higher influence of human activities, while bacterial genera associated with humans were relatively lower in CUL. Both collection sites of die-off samples (Punta Melones in Culebra and Escambrón in San Juan) have inadequate management of wastewater systems resulting in fecal pollution, with negative consequences on the health of marine communities (Norat et al., 2009; Laureano-Rosario et al., 2017). However, CUL is part of a marine-protected area with ecologically friendly recreational activity. ESC is a semi-confined beach in the middle of a metropolitan area and receives a major influence of snorkeling, diving, and other recreational activities being a popular tourist site year-long. In addition, a study has summarized the potential influence of stormwater outfall, the Rio Grande de Loíza River San Juan Bay Estuary, and occasionally outfalls from the Bayamón and Puerto Nuevo Regional Wastewater Treatment Plants (Laureano-Rosario et al., 2017).

Consequently, genera such as *Staphylococcus*, *Streptococcus*, and *Akkermansia* or *Prevotella* which are common human skin and gut bacteria, respectively, were more abundant in *D. antillarum* samples collected from ESC. These human taxa are not commonly detected by fecal water contamination monitoring studies of the area which focus mainly on cultivated Enterococci (Laureano-Rosario et al., 2017). A similar finding in terrestrial wild animal taxa comparing rural to urban wildlife found signs of humanization in anthropized environments with taxa spilling from human activities to the wildlife microbiota (Dillard et al., 2022).

In addition, there was a loss of characteristic taxa in healthy echinoderms, *that is*, *Prolixibacter*, *Propionigenium*, and *Photobacterium* (Yao et al., 2019). In addition to anthropogenic impacts, the shift in the gut microbiota of *D. antillarum* microbial profiles could be explained by the disease stage, that is, those diseased individuals with gut microbiota profiles closer to healthy individuals (i.e., pre-die-off individuals) might be at an earlier disease stage than those individuals with microbiota profiles far apart from healthy individuals.

The absence or the reduction in the abundance of keystone bacterial groups in diseased individuals was more a direct consequence of the pathogenic scuticociliate *Philaster apodigitiformis* than a cause of a pre-disease susceptibility immunological condition.

The gut microbiome composition of *D. antillarum* before the die-off concurs with previous studies of sea urchin microbiota, with *Propionigenium*, *Proxilibacter*, and *Photobacterium* as the most prevalent bacterial genera in the gut (Yao et al., 2019; Rodríguez-Barreras et al., 2021). The occurrence of *Propionigenium*, a propionate-producing bacteria, has been proposed as a stress-response signal (Yao et al., 2019). *Propionigenium* is typically known as a beneficial bacteria that promotes individual health (Bugaut and Bentéjac, 1993; Hosseini et al., 2011; Lee and Hase, 2014). However, its presence as a response to stressful conditions may be due to its ability to help maintain a balanced microbiome even under challenging circumstances. This may suggest that *D. antillarum* samples collected before the die-off event were already under stressful conditions. However, despite these conditions, the etiological agent in animals found in pre-die-off caused uneven demographic consequences with different mortality rates in Puerto Rico (Rodríguez-Barreras et al., 2023b).

To conclude, using new molecular technologies such as 16S rRNA sequencing has significantly enhanced ecological studies in microbiology and our comprehension of ecology, physiology, and interactions within marine ecosystems (Rodríguez-Barreras, 2024). High-throughput metagenomic studies have significantly broadened our understanding of diseases in marine animals, revealing the intricate microbial communities associated with health and disease states in these organisms, which was impossible to do in the 80s. This study, although limited in sample size, as well as not including another multi-kingdom such as eukaryotes, does, however, reveal a dramatic community shift in bacterial diversity and composition in the gut microbiome of *D. antillarum* during the 2022 die-off event in Puerto Rico and perhaps in other jurisdictions along the Caribbean basin. The core microbiome changed between the two groups, indicating a significant restructuring of the sea urchin gut prokaryote community. The described microbiota dysbiosis might be linked with the presence of the pathogenic scuticociliate species *Philaster apodigitiformis* and, certainly, to human-derived activities such as sewage water pollution. Finally, these findings provide valuable insights into the complex interactions between sea urchins, their microbiota, and environmental factors during disease events, highlighting the need for further research into the ecological implications of these microbial changes.

## Data availability statement

The datasets generated for this study can be found in the European Nucleotide Archive Project (ENA) with access numbers EBI: ERP155238 (2022 animals) and ERP123720 (2019 animals).

## Ethics statement

The animal study was approved by University of Puerto Rico Medical Sciences Campus IACUC protocol (IACUC #: A5301118). The study was conducted in accordance with the local legislation and institutional requirements.

## Author contributions

JR-B: Methodology, Writing – review & editing, Writing – original draft, Visualization, Software, Investigation, Formal analysis, Data curation. EK: Validation, Writing – review & editing, Writing – original draft, Visualization, Software, Methodology, Investigation, Formal analysis, Data curation. RR-B: Resources, Writing – review & editing, Writing – original draft, Methodology, Investigation. MQ-O: Visualization, Formal analysis, Writing – review & editing, Writing – original draft, Investigation. CR-D: Funding acquisition, Resources, Project administration, Methodology, Writing – review & editing, Writing – original draft, Investigation. CT-H: Funding acquisition, Writing – review & editing, Writing – original draft, Resources, Project administration, Methodology, Investigation. FG-V: Visualization, Validation, Supervision, Software, Formal analysis, Data curation, Conceptualization, Writing – review & editing, Writing – original draft, Resources, Project administration, Investigation, Funding acquisition.

## Footnotes

^1^https://earthmicrobiome.org/protocols-and-standards/16s/

## Supplementary material

SUPPLEMENTARY FIGURE S1 Rarefaction curves at different levels of rarefaction: without rarefaction **(A)**, rarefaction at 923 reads **(B)**, and rarefaction at 539 reads **(C)**.

SUPPLEMENTARY FIGURE S2 Community diversity using a non-rarefied analyses. Diversity plots and taxonomic composition of *Diadema antillarum*’s gut bacterial communities in 2019 (healthy, pre die-off) and 2022 (healthy and diseased during the die-off) **(A)**. Beta-diversity Non-metric multidimensional scaling (NMDS) derived from Bray-Curtis distances **(B)**. Alpha-diversity box-plots from Chao1 and Shannon’s indices comparing the three groups of healthy and diseased animals collected in 2019 and 2022 **(C)**. Alpha-diversity box-plots from Chao1 and Shannon’s indices comparing the different sites of collection. Taxonomic barplots at the phylum **(D)** and genus levels **(E)**, panels are dividing the three groups of healthy and diseased animals collected in 2019 and 2022. The reads were rarefied at 539 for this analysis.

SUPPLEMENTARY FIGURE S3 Community diversity analyses using a rarefication level of 539 reads. Diversity plots and taxonomic composition of *Diadema antillarum* gut bacterial communities in 2019 (healthy, pre die-off) and 2022 (healthy and diseased during the die-off). Beta-diversity non-metric multidimensional scaling (NMDS) derived from Bray–Curtis distances **(A)**. Alpha-diversity box-plots from Chao1 and Shannon’s indices comparing the three groups of healthy and diseased animals collected in 2019 and 2022 **(B)**. Alpha-diversity box-plots from Chao1 and Shannon’s indices comparing the different sites of collection **(C)**. Taxonomic barplots at the phylum **(D)** and genus levels **(E)**. Panels are dividing the three groups of healthy and diseased animals collected in 2019 and 2022; the reads were not rarefied for this analysis.

SUPPLEMENTARY FIGURE S4 Heat tree analysis depicting alterations in microbiota composition between individuals from 2019 (pre die-off) and individuals from 2022 (during die-off). Significantly altered taxa are displayed by name at the corresponding node. Nodes indicate the hierarchical structure of taxa. A red branch indicates an increase in specific taxa from 2022 compared to controls, while a blue branch indicates a decrease.

SUPPLEMENTARY FIGURE S5 Heatmap of the different bacterial genus with their inferred phylogenetic relationships, per health status and collection sites during the die-off **(A)**. Beta-diversity analysis difference using PERMANOVA to assess the differences between collection sites during the die-off (*p*-value=0.3333) **(B)**.

SUPPLEMENTARY FIGURE S6 Boxplots showing the significant differences in bacterial functional activity between the different collection sites (CAT for Cataño, CGD for Cerro Gordo, LUQ for Luquillo, CUL for Culebra, ESC for Escambron) per year (2019, pre-die-off and 2022, during die-off), using Functional Activity of Prokaryote taxa (FAPROTAX) database **(A–H)**. The functions shown are those who showed a standard deviation higher than 0.01 when comparing the relative abundance of functional active taxa between samples.

SUPPLEMENTARY TABLE S1 Description of the 23 collected samples used in this study with average sample reads and OTUs, per site, and pre-die-off (2019) vs. die-off (2022).

SUPPLEMENTARY TABLE S2 Pairwise *p*-values of alpha diversities values using Chao1 and Shannon pre-die-off (2019) vs. die-off (2022).

SUPPLEMENTARY TABLE S3 Pairwise *p*-values of alpha diversity estimates using Chao1 and Shannon according to collection sites.
