# Supplementary figures and images for: Shifts in the gut microbiota of sea urchin Diadema antillarum associated with the 2022 disease outbreak
Source: Front Microbiol. 2024 Jul 29;15:1409729. doi: 10.3389/fmicb.2024.1409729 (PMC11317302; doi:10.3389/fmicb.2024.1409729)

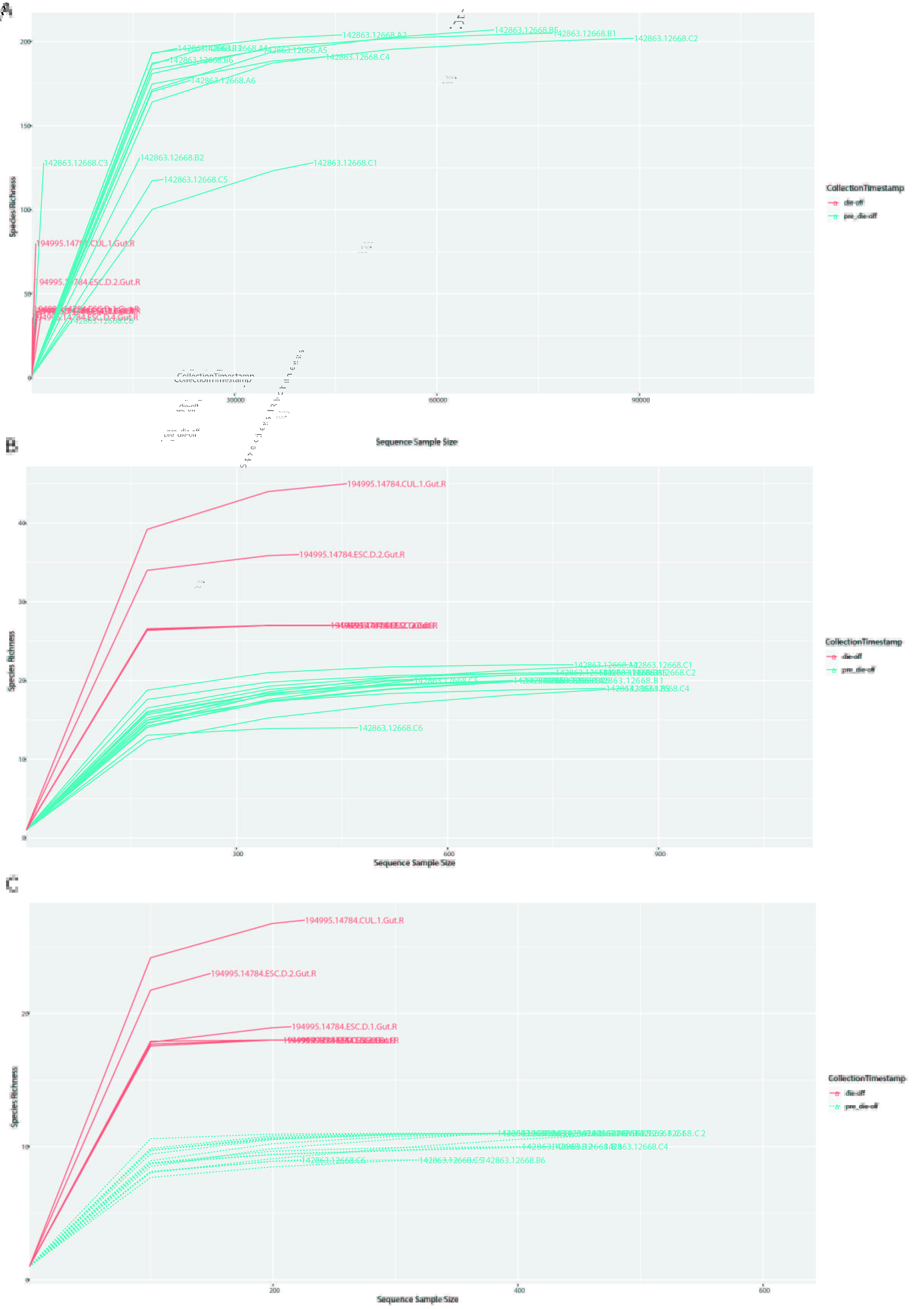

Supplement: SUPPLEMENTARY FIGURE S1 — Rarefaction curves at different levels of rarefaction: without rarefaction (A), rarefaction at 923 reads (B), and rarefaction at 539 reads (C). [file Image_1.JPEG]

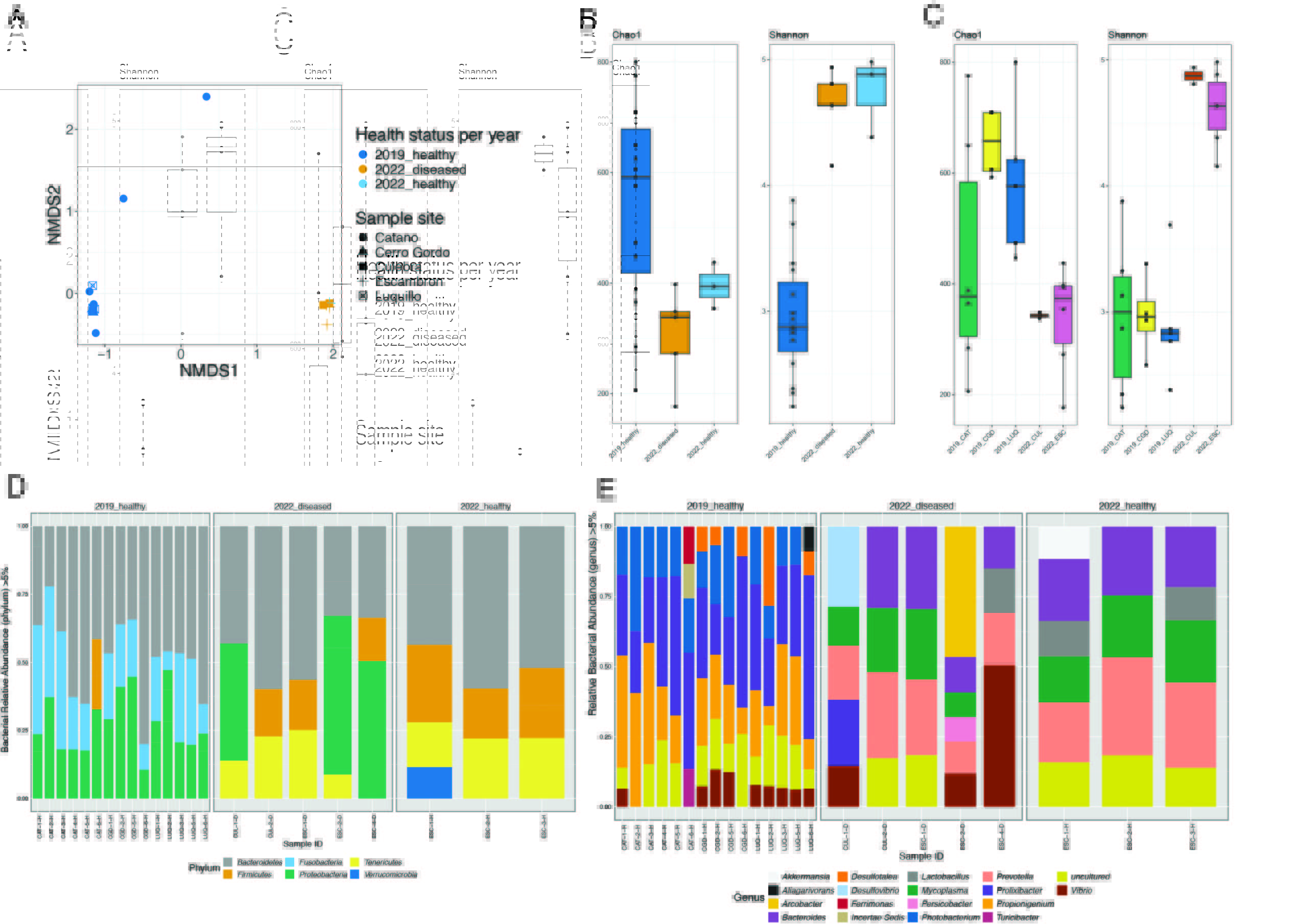

Supplement: SUPPLEMENTARY FIGURE S2 — Community diversity using a non-rarefied analyses. Diversity plots and taxonomic composition of Diadema antillarum’s gut bacterial communities in 2019 (healthy, pre die-off) and 2022 (healthy and diseased during the die-off) (A). Beta-diversity Non-metric multidimensional scaling (NMDS) derived from Bray-Curtis distances (B). Alpha-diversity box-plots from Chao1 and Shannon’s indices comparing the three groups of healthy and diseased animals collected in 2019 and 2022 (C). Alpha-diversity box-plots from Chao1 and Shannon’s indices comparing the different sites of collection. Taxonomic barplots at the phylum (D) and genus levels (E), panels are dividing the three groups of healthy and diseased animals collected in 2019 and 2022. The reads were rarefied at 539 for this analysis. [file Image_2.JPEG]

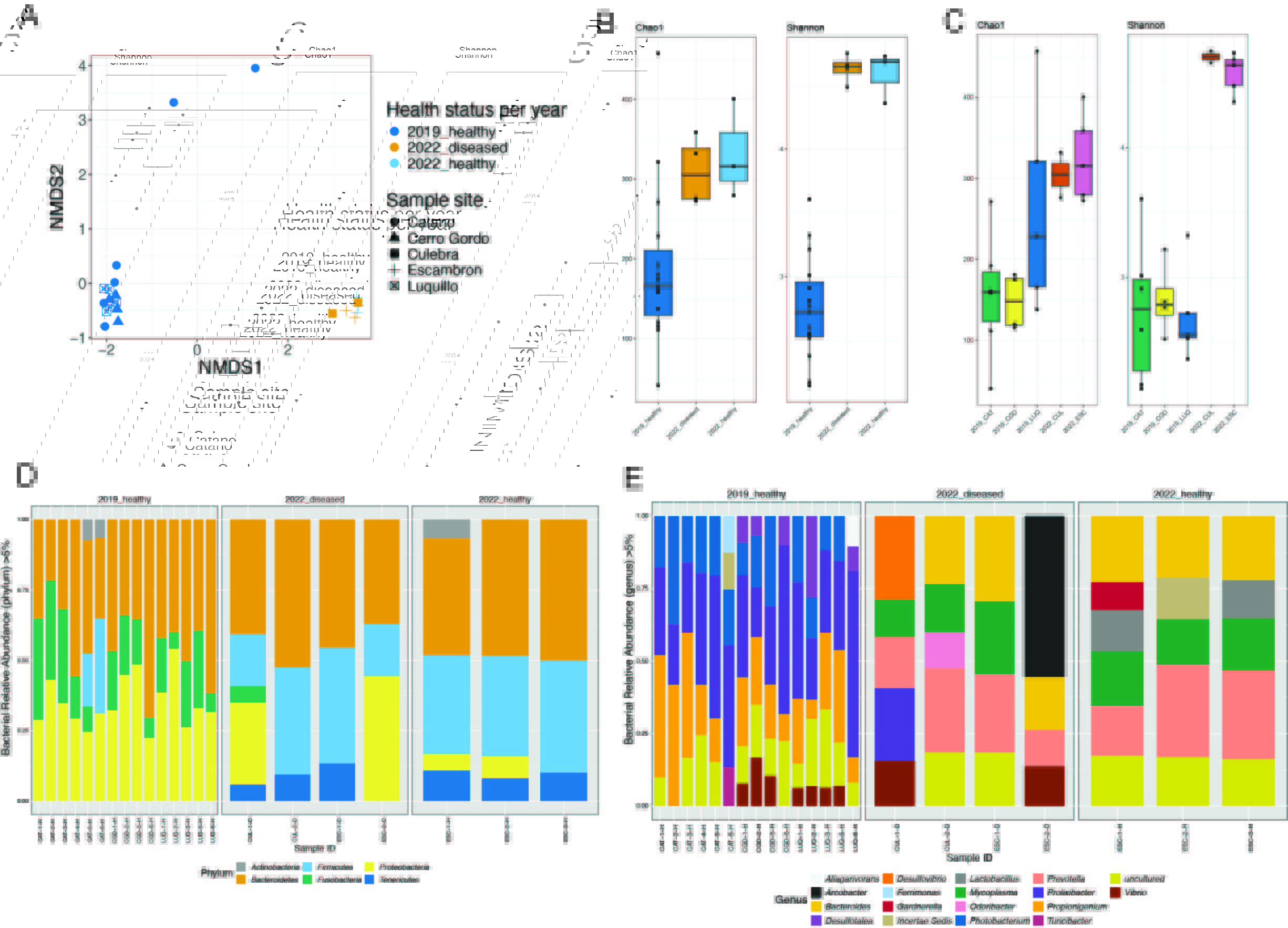

Supplement: SUPPLEMENTARY FIGURE S3 — Community diversity analyses using a rarefication level of 539 reads. Diversity plots and taxonomic composition of Diadema antillarum gut bacterial communities in 2019 (healthy, pre die-off) and 2022 (healthy and diseased during the die-off). Beta-diversity non-metric multidimensional scaling (NMDS) derived from Bray–Curtis distances (A). Alpha-diversity box-plots from Chao1 and Shannon’s indices comparing the three groups of healthy and diseased animals collected in 2019 and 2022 (B). Alpha-diversity box-plots from Chao1 and Shannon’s indices comparing the different sites of collection (C). Taxonomic barplots at the phylum (D) and genus levels (E). Panels are dividing the three groups of healthy and diseased animals collected in 2019 and 2022; the reads were not rarefied for this analysis. [file Image_3.JPEG]

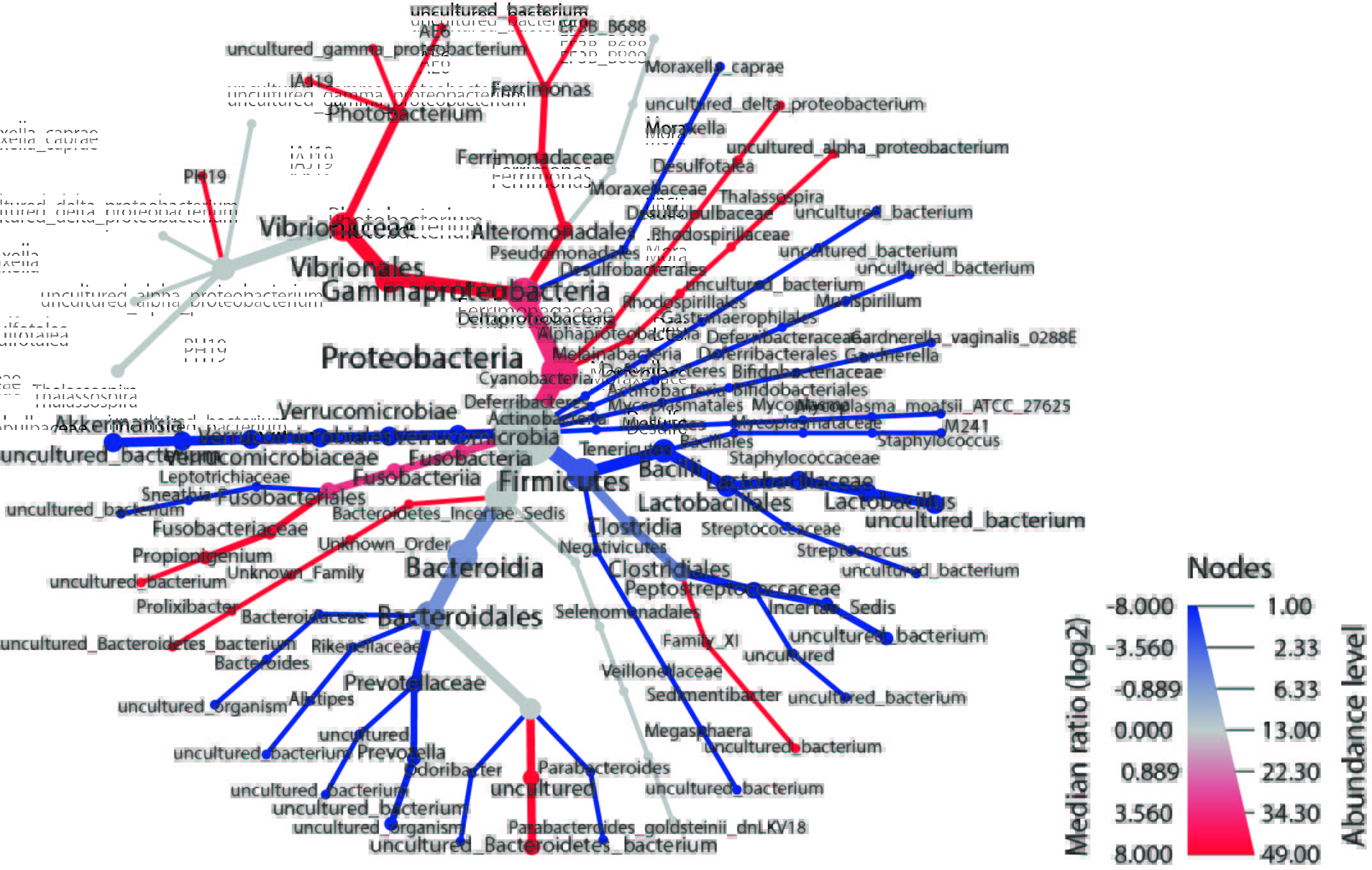

Supplement: SUPPLEMENTARY FIGURE S4 — Heat tree analysis depicting alterations in microbiota composition between individuals from 2019 (pre die-off) and individuals from 2022 (during die-off). Significantly altered taxa are displayed by name at the corresponding node. Nodes indicate the hierarchical structure of taxa. A red branch indicates an increase in specific taxa from 2022 compared to controls, while a blue branch indicates a decrease. [file Image_4.JPEG]

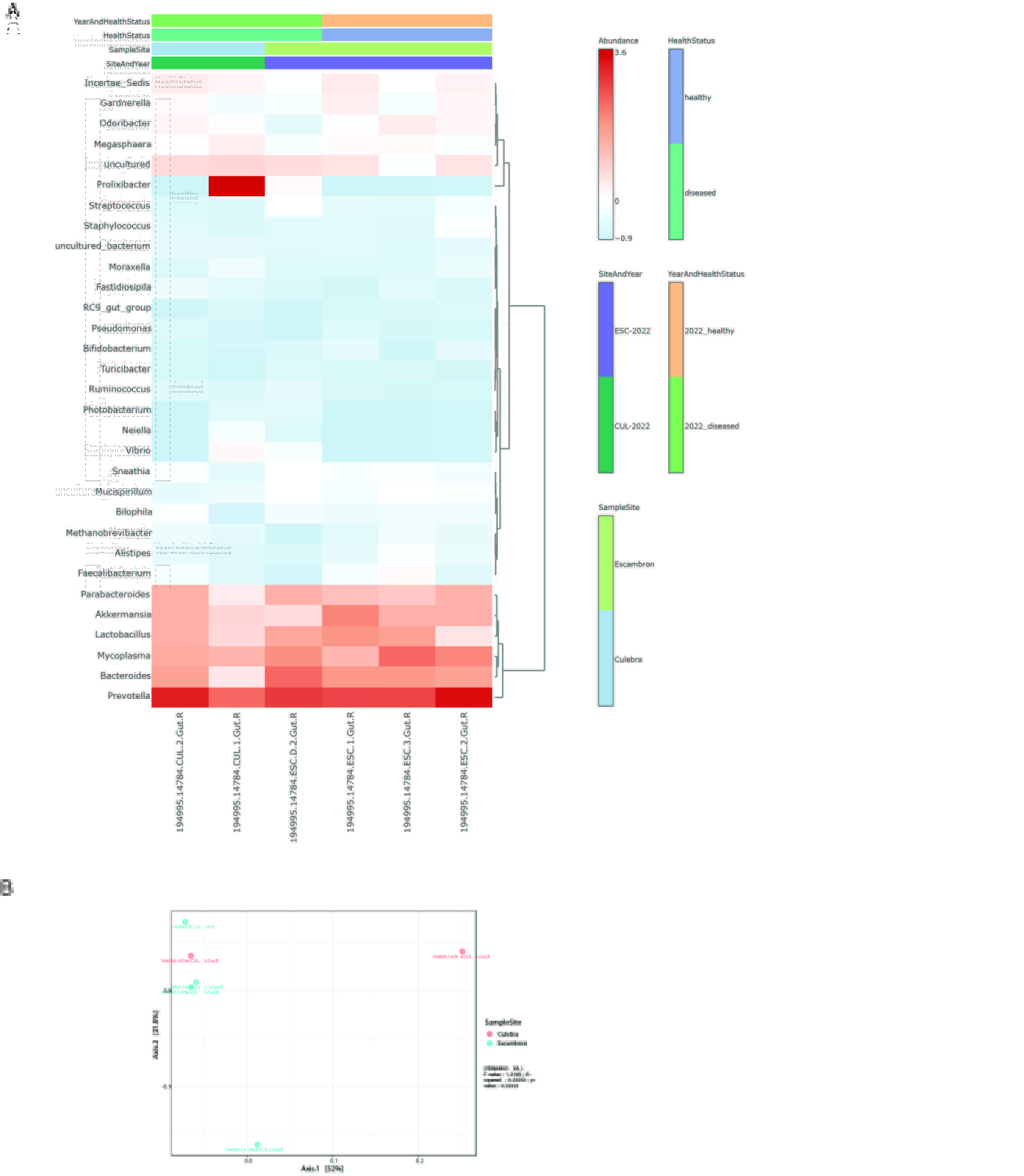

Supplement: SUPPLEMENTARY FIGURE S5 — Heatmap of the different bacterial genus with their inferred phylogenetic relationships, per health status and collection sites during the die-off (A). Beta-diversity analysis difference using PERMANOVA to assess the differences between collection sites during the die-off (p-value=0.3333) (B). [file Image_5.JPEG]

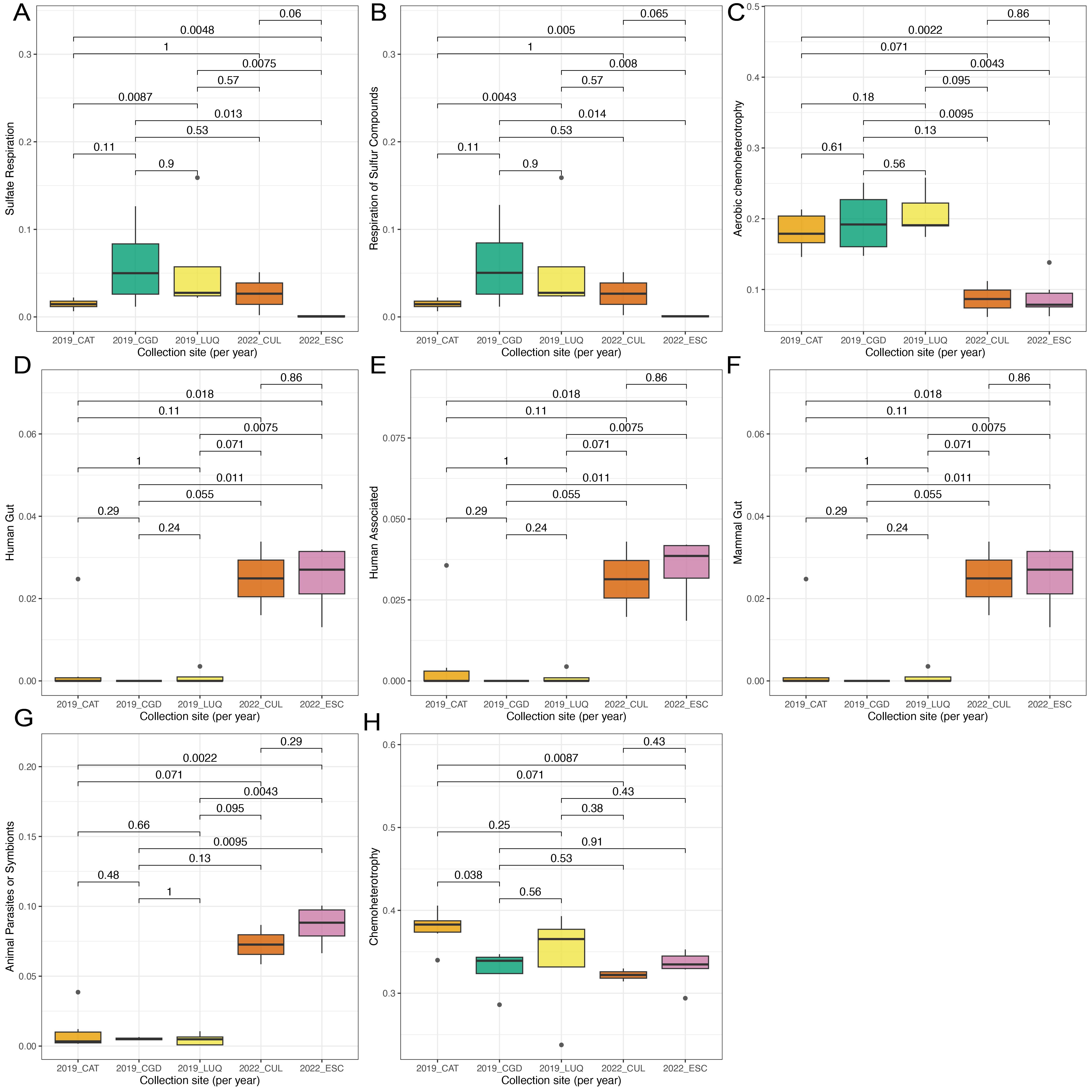

Supplement: SUPPLEMENTARY FIGURE S6 — Boxplots showing the significant differences in bacterial functional activity between the different collection sites (CAT for Cataño, CGD for Cerro Gordo, LUQ for Luquillo, CUL for Culebra, ESC for Escambron) per year (2019, pre-die-off and 2022, during die-off), using Functional Activity of Prokaryote taxa (FAPROTAX) database (A–H). The functions shown are those who showed a standard deviation higher than 0.01 when comparing the relative abundance of functional active taxa between samples. [file Image_6.JPEG]
