# Supplementary material for: Shifts in the gut microbiota of sea urchin Diadema antillarum associated with the 2022 disease outbreak
Source: Front Microbiol. 2024 Jul 29;15:1409729. doi: 10.3389/fmicb.2024.1409729 (PMC11317302; doi:10.3389/fmicb.2024.1409729)
Supplement: SUPPLEMENTARY TABLE S1 — Description of the 23 collected samples used in this study with average sample reads and OTUs, per site, and pre-die-off (2019) vs. die-off (2022). [file Table_1.docx]

| **Site** | **Year** | **Health Status** | ***n*** | **Ave. reads** | **Ave. OTUs** |
| --- | --- | --- | --- | --- | --- |
| Cerro Gordo (CGD) | 2019 | healthy | 4 | 33,814.5 ± 10,188.73 | 489 ± 102.83 |
| Luquillo (LUQ) | 2019 | healthy | 5 | 42,347.8 ± 30,281.12 | 522.2 ± 141.77 |
| Catano (CAT) | 2019 | healthy | 6 | 36,010 ± 31,644.29 | 373 ± 177.72 |
| Culebra (CUL) | 2022 | diseased | 2 | 1,878 ± 1,243.09 | 305 ± 72.12 |
| Escambron (ESC) | 2022 | diseased | 3* | 567.66 ± 341.90 | 167.33 ± 60.70 |
| Escambron (ESC) | 2022 | healthy | 3 | 1,449 ± 241.42 | 284.66 ± 18.01 |
| * two animals had lower than 923 reads |  |  |  |  |  |

**Supplementary Table 1.** Description of the 23 collected samples used in this study with average sample reads and OTUs, per site, and pre die-off (2019) *vs*. die-off (2022).
