# Supplementary material for: Shifts in the gut microbiota of sea urchin Diadema antillarum associated with the 2022 disease outbreak
Source: Front Microbiol. 2024 Jul 29;15:1409729. doi: 10.3389/fmicb.2024.1409729 (PMC11317302; doi:10.3389/fmicb.2024.1409729)
Supplement: SUPPLEMENTARY TABLE S2 — Pairwise p-values of alpha diversities values using Chao1 and Shannon pre-die-off (2019) vs. die-off (2022). [file Table_2.docx]

**Supplementary Table 2.** Pairwise p-values of alpha diversities values using Chao1 and Shannon pre-die-off (2019) *vs*. die-off (2022).

| **Shannon's index** | | | |
| --- | --- | --- | --- |
| **All groups** | **H** | **p-value** |  |
|  | 12.3117 | 0.0021 |  |
|  |  |  |  |
| **Pair** | **H** | **p-value** | **q-value** |
| 2019_healthy vs 2022_diseased | 7.1053 | 0.0077 | 0.0115 |
| 2019_healthy vs 2022_healthy | 7.1053 | 0.0077 | 0.0115 |
| 2022_diseased vs 2022_healthy | 0.4286 | 0.5127 | 0.5127 |
|  |  |  |  |
| **Chao's index** |  |  |  |
|  |  |  |  |
| **All groups** | **H** | **p-value** |  |
|  | 10.1922 | 0.0061 |  |
|  |  |  |  |
| **Pair** | **H** | **p-value** | **q-value** |
| 2019_healthy vs 2022_diseased | 7.1053 | 0.0076 | 0.0231 |
| 2019_healthy vs 2022_healthy | 3.8211 | 0.0506 | 0.0506 |
| 2022_diseased vs 2022_healthy | 3.8571 | 0.0495 | 0.0506 |
|  |  |  |  |
