# Supplementary material for: Shifts in the gut microbiota of sea urchin Diadema antillarum associated with the 2022 disease outbreak
Source: Front Microbiol. 2024 Jul 29;15:1409729. doi: 10.3389/fmicb.2024.1409729 (PMC11317302; doi:10.3389/fmicb.2024.1409729)
Supplement: SUPPLEMENTARY TABLE S3 — Pairwise p-values of alpha diversity estimates using Chao1 and Shannon according to collection sites. [file Table_3.docx]

| **Site** | **Year** | **Health Status** | ***n*** | **Ave. reads** | **Ave. OTUs** |
| --- | --- | --- | --- | --- | --- |
| Cerro Gordo (CGD) | 2019 | healthy | 4 | 33,814.5 ± 10,188.73 | 489 ± 102.83 |
| Luquillo (LUQ) | 2019 | healthy | 5 | 42,347.8 ± 30,281.12 | 522.2 ± 141.77 |
| Catano (CAT) | 2019 | healthy | 6 | 36,010 ± 31,644.29 | 373 ± 177.72 |
| Culebra (CUL) | 2022 | diseased | 2 | 1,878 ± 1,243.09 | 305 ± 72.12 |
| Escambron (ESC) | 2022 | diseased | 3* | 567.66 ± 341.90 | 167.33 ± 60.70 |
| Escambron (ESC) | 2022 | healthy | 3 | 1,449 ± 241.42 | 284.66 ± 18.01 |
| * two animals had lower than 923 reads |  |  |  |  |  |

**Supplementary Table 3.** Pairwise p-values of alpha diversity estimates using Chao1 and Shannon according to collection sites.

|  |  |  |  |
| --- | --- | --- | --- |
| **All groups** | **H** | **p-value** |  |
|  | 12.7130 | 0.0128 |  |
|  |  |  |  |
| **Pair** | **H** | **p-value** | **q-value** |
| 2019_Catano vs 2019_Cerro Gordo | 0.0000 | 1.0000 | 1.0000 |
| 2019_Catano vs 2022_Culebra | 4.0000 | 0.0455 | 0.1056 |
| 2019_Catano vs 2022_Escambron | 6.5455 | 0.0105 | 0.0697 |
| 2019_Catano vs 2019_Luquillo | 0.0000 | 1.0000 | 1.0000 |
| 2019_Cerro Gordo vs 2022_Culebra | 3.4286 | 0.0641 | 0.1068 |
| 2019_Cerro Gordo vs 2022_Escambron | 5.3333 | 0.0209 | 0.0697 |
| 2019_Cerro Gordo vs 2019_Luquillo | 2.1600 | 0.1416 | 0.2023 |
| 2022_Culebra vs 2022_Escambron | 0.2143 | 0.6434 | 0.8043 |
| 2022_Culebra vs 2019_Luquillo | 3.7500 | 0.0528 | 0.1056 |
| 2022_Escambron vs 2019_Luquillo | 6.0000 | 0.0143 | 0.0697 |
|  |  |  |  |
| **Chao's index** |  |  |  |
|  |  |  |  |
| **All groups** | **H** | **p-value** |  |
|  | 10.3130 | 0.0355 |  |
|  |  |  |  |
| **Pair** | **H** | **p-value** | **q-value** |
| 2019_Catano vs 2019_Cerro Gordo | 0.0455 | 0.8312 | 0.8312 |
| 2019_Catano vs 2022_Culebra | 4.0000 | 0.0455 | 0.1282 |
| 2019_Catano vs 2022_Escambron | 3.6818 | 0.0550 | 0.1282 |
| 2019_Catano vs 2019_Luquillo | 0.8333 | 0.3613 | 0.4015 |
| 2019_Cerro Gordo vs 2022_Culebra | 3.4286 | 0.0641 | 0.1282 |
| 2019_Cerro Gordo vs 2022_Escambron | 5.3333 | 0.0209 | 0.1282 |
| 2019_Cerro Gordo vs 2019_Luquillo | 0.9600 | 0.3272 | 0.4015 |
| 2022_Culebra vs 2022_Escambron | 0.8571 | 0.3545 | 0.4015 |
| 2022_Culebra vs 2019_Luquillo | 3.7500 | 0.0528 | 0.1282 |
| 2022_Escambron vs 2019_Luquillo | 2.9400 | 0.0864 | 0.1440 |
|  |  |  |  |
